# The Price of the Publican

**Published:** 1895-02-23

**Authors:** 


### Modern Sociology.

### THE PRICE OF THE PUBLICAN.

In temperance legislation compensation blocks the way. Almost everybody is agreed that there are too many public-houses in most places, and that some ought to be suppressed. But which, and on what terms? Why should Brown's tavern be suppressed and Smith's allowed to flourish—to flourish all the more that the rival house is shut, and Brown's customers now go to Smith for their liquor? To those who think that Smith, Brown, and *hoc genus omne* are unmitigated public evils the answer is easy, but these are, and promise long to remain, in a minority, and practical people, who do not want to defer dealing with drunkenness and its causes till the millennium, are seeking for a *via media* that will diminish temptation without ignoring the rights of the publican.

The publican has rights; that is to be remembered. It may be said that he does not pay for his license, and therefore has no property in it. But when he bought the goodwill of the public-house he occupies he paid a considerable sum for it on the assumption that the license would be renewed. He pays a higher rent than he would for a shop of the same size which had no license attached to it. He may, therefore, reasonably contend that the license is a property which he has paid, and is paying for, and which he should not be deprived of unless it can be shown that he has misused the privileges it gives. If, however, it is felt to be for the public interest that his house should be shut up, the public interest must be consulted. Still, it is contrary to all the principles of English justice that the private individual should be wholly sacrificed. When a new street has to be cut through a town, or a new railway laid, we have compulsory sale of property, the site of which is required for the new works, but not confiscation. On the contrary, because the sale is forced upon the owner he usually receives a high price for his ground, as compensation for the compulsion. Why should a tradesman, if he has conducted his business honestly and respectably, be treated with less consideration?

The parallel case of the compensation paid to slave-owners has been quoted *ad nauseam*, yet its bearing on the matter of the publican and his claims is too pertinent to permit of its being ignored. The nation gave a large sum to free the slaves in our colonies, and it has been accounted to us for righteousness. To free the slaves—that is, to compensate the slave-owners who, either by purchase or inheritance, had become possessed of these living chattels—the English nation, less introspective and hair-splitting in its mental habit then than now, once convinced that slave-owning was cruel and unchristian, took the direct and practical method of ending it, undeterred by the fear that in doing a great good to the slave they might do a little good to the slave-owner. But on the other side it must be remembered that the good thus done was small. Only, on an average, half the market value of a slave was given, or even less. Some figures quoted by the Bishop of Chester, in his "Chart of Compensation," show this. In Bermuda the average sale value of a slave was £27 4s. 11d., and the rate of compensation was £12 10s. 5d. In Barbadoes the value was £47 1s. 3d., and the compensation £20 13s. 8d. In Cape Colony the figures stand at £73 9s. 11d. and £34 11s. 7d. respectively. Thus it can hardly be said that the slave-owners made a profit out of the national conscience, even if we ignore the great and even fatal blow dealt to the industries of the colonies.

There is, therefore, no precedent for compensation on a lavish scale. And, indeed, were the nation determined to compensate the publicans at the same rate as the slave-owners, it would deal too generously if it gave them half the stated value of their houses. It is not thinking too meanly of human nature to surmise that where they hope to dip their hands in the national purse they will extend them to the utmost. Property and business alike will be estimated at the highest value possible, till the world will wonder, not that some brewers are rich, but that every bar-tender is not a millionaire. It would be wise in these cases to take the advice of Mr. Gilbert's cantankerous king and compare these estimates with the income tax returns; and also with the valuations offered to the Probate Court. Even where a large sum has been paid for a licensed house within recent years, justice and common sense could refuse to accept this as a true valuation, for when first the suppression of licenses was spoken of, compensation with a free hand was generally taken for granted, and hence arose much speculation in public-houses—a speculation which we need not regret threatens to turn out unprofitable. The "tied-houses" in connection with breweries also have little claim to compensation. The tied-house system means that the wholesale trade controls the retail, that, in short, liquor-selling becomes the monopoly of a gigantic and powerful ring; and it is to be feared that brewery and distillery companies, by means of their superior wealth and influence, can often obtain the renewal of a license where it would be refused to a private trader, or even get a new license granted for one of their houses while a sop is thrown to the temperance Cerberus by the concurrent suppression of some other public-house. The means taken by some companies to compel a market for their wares are, indeed, an interference with free trade, unjust to the man who nominally holds the license (and who will be the person to suffer for any faults in the management of the house), but who is really a servant of the company; and unjust also to the public, for the true lessee of the house often charges the publican a lower rent than he himself pays, counting on the profit from the sale of his beer, and thus causing the property to be rated lower than it would otherwise be—a distinct injustice to the ratepayers.

Thus the case stands that, though the publican has some claim to have the loss he undergoes by the loss of his license made good to him, it does not follow that he has a right, either moral or legal, to get all he asks for. He is no public benefactor, to be specially considered in parting with him; and it would need a commission of careful, impartial, and experienced men to analyse his claims, and see how far they are just and how far they are manufactured with a view to obtaining an undue share of public conscience-money.

